# Stability and Turnover of the ACTH Receptor Complex

**DOI:** 10.3389/fendo.2019.00491

**Published:** 2019-07-26

**Authors:** Adrian J. L. Clark, Li Chan

**Affiliations:** Barts and the London School of Medicine and Dentistry, Centre for Endocrinology, William Harvey Research Institute, Queen Mary University of London, London, United Kingdom

**Keywords:** ACTH receptor, adrenal cortex, glucocorticoid, G protein-coupled receptor, adrenocorticotropin, melanocortin receptor

## Abstract

Glucocorticoid production in mammals is principally regulated by the action of the pituitary hormone adrenocorticotropin (ACTH) acting on its cognate membrane receptor on the zona fasciculata cells of the adrenal cortex. The receptor for ACTH consists of two essential components, a small seven transmembrane domain G protein-coupled receptor of the melanocortin receptor subgroup known as the melanocortin 2 receptor (MC2R) and a small single transmembrane domain protein that adopts a antiparallel homodimeric form and which is known as the melanocortin 2 receptor accessory protein (MRAP). MRAP is essential for the trafficking of the MC2R to the cell surface as well as being required for receptor responsiveness to ACTH at physiological concentrations—probably by facilitating ACTH binding, but possibly also by supporting G protein interaction with the MC2R. A number of studies have shown that ACTH stimulates the expression of functional receptor at the cell surface and the transcription of both MC2R and MRAP mRNA. However, the time course of these transcriptional effects differs such that MRAP is expressed relatively rapidly whereas MC2R transcription responds much more slowly. Furthermore, recent data suggests that MRAP protein is turned over with a short half-life whereas MC2R has a significantly longer half-life. These findings imply that these two ACTH receptor proteins have distinct trajectories and that it is likely that MRAP-independent MC2R is present at the cell surface. In such a situation newly transcribed and translated MRAP could enable the rapid recruitment of functional receptor at the plasma membrane without the need for new MC2R translation. This may be advantageous in circumstances of significant stress in that the potentially complex and perhaps inefficient process of *de novo* MC2R translation, folding, post-translational modification and trafficking can be avoided.

The pituitary-adrenal axis provides a vital mechanism for protection against stress, allowing higher centers to rapidly generate increases in circulating glucocorticoids. These in turn adjust the metabolic and immunological functions of the body to cope with the altered environment. Failure of this system is potentially fatal. It is surprising therefore that there is not greater redundancy among the components of this system. For example, the key messenger hormone, adrenocorticotropin (ACTH) is the product of a single gene and is secreted from a single population of corticotroph cells in a single anatomical organ—the anterior pituitary gland. ACTH in turn acts through a single receptor located almost entirely on the surface of cells of the two adrenal glands, resulting in the production of a single glucocorticoid hormone—cortisol or corticosterone. Although the ACTH receptor was one of the first receptors to be shown to signal through stimulation of cyclic AMP (1), and one of the first receptors for which ligand binding was demonstrated (2), the molecular characterization of this entity was relatively delayed. Protein purification approaches were largely unsuccessful, and it was not until a gene was identified through reduced stringency PCR which was shown to be closely related to a gene encoding a receptor for α-melanocyte stimulating hormone (α-MSH). This gene was expressed almost exclusively in the adrenal cortex and hence was described as the melanocortin 2 receptor (MC2R) and proposed to encode the ACTH receptor (3). Subsequent demonstration of mutations in this gene in patients with ACTH resistance lent support to this identification (4, 5).

With most receptors for which a gene or cDNA is identified in this way the usual practice is to transfect the candidate DNA into a cell type that lacks the receptor in question and to show the cell acquires the characteristics of the receptor. In the case of the ACTH receptor however this proved very difficult. Some evidence of expression could be found in cell types that already possessed MSH receptors (3, 6), but expression in a true null cell devoid of any MSH receptor was not achievable.

The first evidence that the MC2R required a co-factor came from the demonstration that it could be expressed in adrenal cell lines that lacked endogenous ACTH receptors—implying that there was something else about the adrenal cell required for successful expression (7). However, it was not until a new gene was found that was mutated in patients with ACTH resistance but a normal MC2R gene that a solution to this problem was identified. This new gene encoded a small single transmembrane domain protein that when co-expressed with the MC2R in transfected cells conferred typical ACTH responsiveness to those cells. This gene was named the melanocortin 2 receptor accessory protein—MRAP (8).

Evidence from a number of labs showed that the MRAP protein interacted with the MC2R as a homodimer and then trafficked with the receptor to the plasma membrane (9, 10). This appeared to be the primary function of MRAP in that without MRAP, the MC2R protein accumulated at the ER and did not appear at the cell surface.

At the cell surface MRAP maintained an association with the receptor protein and the evidence suggests it is required for ACTH binding or possibly in signal transduction (11, 12). Perhaps the most unusual phenomenon associated with MRAP is that it exists as an antiparallel homodimer spanning the plasma membrane such that one N-terminus is extracellular and one is intracellular (9). This topology is probably unique in eukaryotic biology, and suggests a very unusual mode of action that remains poorly understood.

These aspects of MRAP have been reviewed elsewhere (13, 14) and are not the prime focus of this paper. Instead, we will consider the dynamic aspects of MRAP which may challenge the notion that it is the “loyal and lifelong” partner of the MC2R as may be assumed, or whether it has a more complex and perhaps transient role with its partner protein.

## Induction of MC2R mRNA by ACTH

It was recognized some years before the ACTH receptor was cloned and characterized that ACTH could induce increased responsiveness to itself and an increased number of receptors in the adrenal. Thus, for example, Durand and Locatelli (15) demonstrated in rabbits that ACTH treatment increased the number of its adrenal receptors. Similar findings were reported from fetal sheep adrenals (16). Penhoat et al. (17) showed that 48 h treatment with physiological concentrations of ACTH increased ACTH ligand binding on bovine adrenal cells four-fold.

Following the cloning of the *MC2R* several groups explored the influence of ACTH and other stimuli on *MC2R* mRNA. Thus Mountjoy et al. (18) showed a 6-fold increase in *Mc2r* mRNA in mouse Y1 cells and a 2-fold increase in human H-295 cells after 24 h of treatment with ACTH. Lebrethon et al. (19) found a 21-fold increase in *MC2R* mRNA and a 4-fold increase in ACTH binding in human primary adrenal cells, initial responses being seen within 12 h of treatment, and similar changes after 48 h treatment with ACTH in human fetal adrenal cells (20). Rehman et al. (21) showed induction of *MC2R* mRNA after ACTH after 6 h in cultured human fetal adrenal cells. More recently Liu et al described the effect of restraint stress or ACTH injection on rat adrenal *Mc2r* mRNA over a short time period and failed to show any changes over the initial 4 h (in response to restraint) or over the first 60 min in response to ACTH (22). These and other similar studies are summarized in Table 1.

**Table 1:** Summary of studies of MC2R and MRAP mRNA expression changes in response to various stimuli.

**Species and model**	**Stimulus**	**Time**	**MC2R mRNA**	**MRAP mRNA**	**References**
Y1 cells	ACTH (10^−8^M)	24 h	6x	nd	Mountjoy et al. (18)
H-295 cells	ACTH (10^−8^M)	24 h	2x	nd	Mountjoy et al. (18)
H-295 cells	Forskolin (10^−5^M)	3 h	1x	nd	Mountjoy et al. (18)
H-295 cells	Forskolin (10^−5^M)	12 h	3x	nd	Mountjoy et al. (18)
H-295 cells	Forskolin (10^−5^M)	24 h	3.5x	nd	Mountjoy et al. (18)
H-295 cells	db cAMP (1mM)	24 h	3x	nd	Mountjoy et al. (18)
Human primary cult	ACTH (10^−8^M)	72 h	21 x	nd	Lebrethon et al. (19)
Human fetal adrenal cells	ACTH (10^−9^M)	48 h	18 x	nd	Lebrethon et al. (20)
Human fetal adrenal cells	ACTH (10^−8^M)	3 h	1x	nd	Rehman et al. (21)
Human fetal adrenal cells	ACTH (10^−8^M)	6 h	3 x	nd	Rehman et al. (21)
Human fetal adrenal cells	ACTH (10^−8^M)	12 h	12x	nd	Rehman et al. (21)
Human fetal adrenal cells	ACTH (10^−8^M)	24 h	18 x	nd	Rehman et al. (21)
Rat adrenal (*in vivo*)	Restraint stress	1–4 h	1x	5x (at 2h)	Liu et al. (22)
Rat adrenal (*in vivo*)	ACTH (5μg i.p.)	0–1 h	1x	1x^*^	Liu et al. (22)
Rat adrenal (*in vivo*)	LPS (25 μg i.v.)	6 h	0.5x	4x	Gibbison et al. (23)
Human fetal adrenal cells	ACTH (10^−8^M)	48 h	11x	8x	Xing et al. (24)
Human adult adrenal cells	ACTH (10^−8^M)	48 h	12x	16x	Xing et al. (24)
Human adult adrenal cells	ACTH (10ng/ml)	48 h	20 x	11 x	Hofland et al. (25)
Human adult adrenal cells	Forskolin (10^−6^M)	48 h	30x	10x	Hofland et al. (25)

* *Increase in MRAP hnRNA by 15-fold at 15 mins*. *nd: not measured*.

## Induction of MRAP mRNA by ACTH

As the *MRAP* gene was only identified in 2005 these earlier studies did not examine its response to ACTH and related stimuli, and it is only in later studies that both *MC2R* and *MRAP* gene expression in the same model has been reported.

Thus in their study Liu et al. (22) demonstrated the rapid induction of adrenal *Mrap* mRNA by about 5-fold within 2 h in their model of restraint stress, and a 15-fold induction of *Mrap* heteronuclear RNA within 15 min of ACTH injection (although no change in mature *Mrap* mRNA was observed in this time frame). These increases were not sustained and in the restraint stress model *Mrap* mRNA had almost normalized after 4 h. Gibbinson et al. (23) found a 4-fold increase in adrenal *Mrap* mRNA 6 h after LPS injection in the rat (at a time when *Mc2r* mRNA levels had fallen).

Over a longer time period Xing et al. (24) used a microarray approach to explore gene expression changes after 48 h of treatment of human fetal or adult adrenals demonstrating a 16-fold and 12-fold change of *MRAP* and *MC2R* expression (respectively) in adult adrenal, and 8-fold and 11-fold increases in these two genes in fetal adrenal. Hofland et al. (25) demonstrated 11 and 20-fold increases in *MRAP* and *MC2R* in human normal adrenal primary cultures exposed to 48 h treatment with ACTH. These various studies are summarized in Table 1.

In summary, although a range of models and stimuli have been used, it is unfortunate that few have examined the response of both *MC2R* and *MRAP* over a time course that encompasses both acute and more delayed time periods. However, it does seem from the available data that while *MC2R* is almost universally induced over a longer time period of 12−24 h or more, *MRAP* is capable of making a more rapid response to ACTH, restraint stress or LPS stimulation.

## ACTH Receptor Proteins

The next question that arises is what happens to the ACTH receptor protein complex after its translation. Very little data has been collected on the behavior of the MC2R and MRAP proteins—in large part because of absence of good quality antibodies.

**Figure 1:**
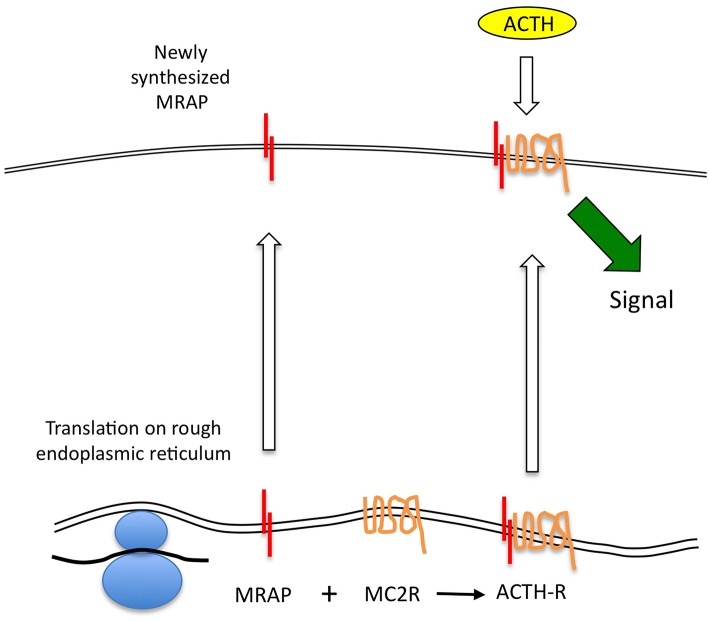
ACTH receptor protein expression. MC2R mRNA is translated at the endoplasmic reticulum (ER), and may independently adopt a correctly folded and orientated receptor conformation. However, it is unable to traffic beyond this point to the plasma membrane. MRAP mRNA is translated and adopts an anti-parallel homodimeric conformation at the ER. This complexes with the MC2R protein and may assist with its correct conformational folding before enabling the complex to traffic to the cell surface. Only the MRAP-MC2R complex is competent to bind ACTH at physiological concentrations and thence to generate a steroidogenic signal.

It seems that the MRAP homodimer forms at the same time as, or very soon after translation, and that the MRAP molecule in which the N-terminus is in the extracellular position is then glycosylated—probably stabilizing the homodimer at this time. This then interacts with the MC2R protein at the level of the endoplasmic reticulum or Golgi (26). This complex is capable of trafficking to the cell surface, although whether MRAP has an additional role in supporting correct folding of the MC2R is not clear. It is also unclear what the fate of un-complexed MC2R is at the ER—for example in circumstances in which MRAP is not expressed. In studies in cells lacking MRAP, transfected MC2R accumulated substantially at the ER, although the relationship of such overexpression systems to physiology is uncertain (27). It is also clear that in the absence of MC2R, MRAP will traffic efficiently to the cell surface (8, 9). The key elements of this conventional model are shown in Figure 1.

## ACTH Receptor Turnover

In an article published in this journal in 2016, Maben et al traced the fate of MRAP in transfected CHO cells (28). They concluded that MRAP has a half-life of 1.7 h and is not stabilized by the presence of MC2R expression. Using similar techniques they estimated that the MC2R protein half-life was between 3 and 8 h. This is a surprising finding as it implies that after reaching the plasma membrane MRAP and MC2R turn over independently of each other. This is apparently in contradiction to the “lifetime partnership” model and implies that MRAP-independent MC2R is likely to be present at the cell surface under most circumstances and is presumably unresponsive to ACTH.

Clearly this half-life data needs replication although it was obtained using a robust but relatively novel biotin-labeling technique as well as by a more traditional cycloheximide technique with similar findings. It has the disadvantage of deriving from transfected CHO cells rather than cells in which MRAP and MC2R were endogenously expressed. Unquantitated data from transfected OS3 adrenocortical cells was also shown in the same paper which appeared to suggest a substantially longer half life for the MRAP protein—particularly in the absence of MC2R. OS3 cells express endogenous and functional MRAP, but no endogenous MC2R (29). Maben et al. argued that OS3 cells grow more slowly than CHO cells and that this may explain the slower turnover, but clearly an alternative explanation is that adrenal cells protect the turnover of these proteins for functional reasons. This question needs further exploration.

**Figure 2:**
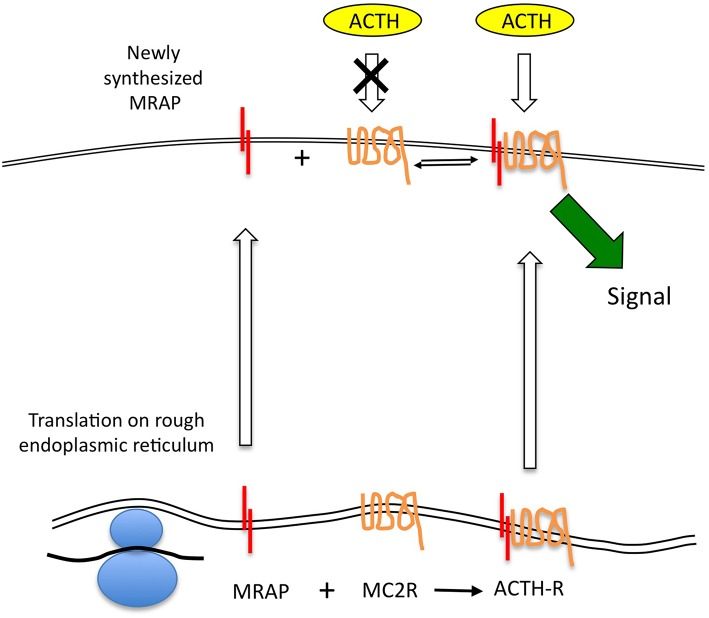
The MRAP-MC2R ACTH receptor complex may be relatively labile allowing MRAP protein to turnover more quickly than the MC2R, and thus leaving uncomplexed MC2R, which is believed to be unresponsive to ACTH stimulation, at the cell surface. This open the possibility that newly transcribed MRAP mRNA can be quickly translated and traffic to the cell surface to complex with “free” MC2R. Such a model permits a rapid response in the form of new signaling-competent receptor recruitment.

If we accept that MRAP protein does turnover rapidly and at a faster rate than MC2R, it seems probable that the ACTH receptor complex is not a tight one and that free MRAP (presumably in its homodimeric form) and MC2R may be present at the cell surface. Unfortunately there is no published data on the relative quantities of these two proteins in adrenal cells expressing functional ACTH receptors. There is also no data on the affinities of the MRAP dimer for the receptor. Roy et al provided immunofluorescent imaging data to support the existence of non-complexed MC2R and MRAP in transfected cells (30). If there is free MC2R on the cell surface this would form a reservoir of “spare” receptor that was incapable of responding to ACTH. Consequently, the relatively rapid transcription of *MRAP* in response to the various stimuli described earlier may enable functionally “new” receptor to be recruited *at the cell surface* in the absence of rapid *MC2R* gene expression. This would provide an attractive and efficient mechanism for the rapid upregulation of the ACTH receptor without having to depend on the accurate folding of new MC2R protein. Elements of this model are shown in Figure 2.

**Figure 3:**
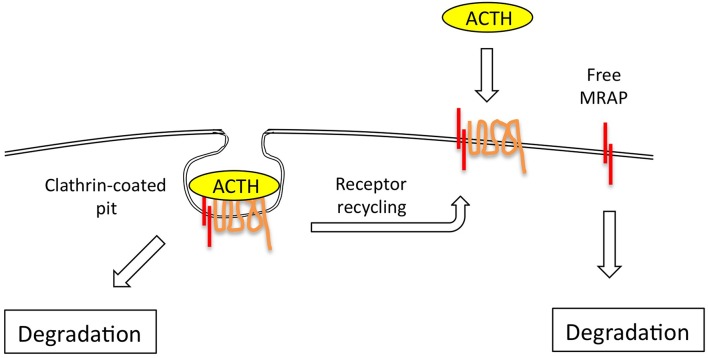
The ACTH receptor complex internalizes via clathrin-coated pits, following which receptor is either rapidly re-cycled to the plasma membrane, or degraded. The evidence suggests that MRAP is also independently and fairly rapidly degraded via ubiquitin-dependent and independent mechanisms.

The ACTH receptor desensitizes and internalizes after agonist stimulation via clathrin-coated pits and re-cycles directly, or via the Golgi-recycling pathway (31, 32). Around 70% of the cell surface receptor that binds ACTH does not internalize however (31, 32) (Figure 3), and it would be interesting to know if this is the un-partnered MC2R that was unable to signal. This may be a false conclusion however as Sebag and Hinkle (11) have shown that MRAP determines ACTH ligand binding via a specific LDYI motif in the N-terminal region, so un-partnered MC2R may be unlikely to bind the ACTH tracer used in these studies. This situation may be more complex however in that MRAP2, a paralogue of MRAP, which lacks the critical LDYI ACTH binding motif of MRAP will support ACTH signaling, and hence binding, at high (micromolar) ACTH concentrations (33).

Other than the adrenal cortex, MRAP is also expressed in adipocytes, and indeed was first identified as a protein of unknown function that was expressed as 3T3-L1 fibroblasts differentiated into adipocytes (34). Zhang et al. (35) have shown in differentiated 3T3-L1 adipocytes that the N-terminus of MRAP interacts with the Gα_s_ protein and that the region between residues 14 and 17 of MRAP is critical for this role. While these findings may be compatible with each other, Sebag and Hinkle had previously demonstrated that the MRAP 14-17 deletion mutant was fully capable of signaling in their earlier studies (11).

## Conclusions

MRAP is a very unusual protein that appears to have a critical role in permitting the MC2R to traffic to the cell surface and to respond to ACTH—either by influencing ACTH ligand binding or by approximating the Gα_s_ protein to the receptor (or both). ACTH receptor expression can be stimulated by a variety of exogenous stimuli including ACTH exposure. This is mediated by the relatively rapid increase of MRAP transcription while MC2R transcription is more delayed. Newly translated MRAP will enable any MC2R still resident at the endoplasmic reticulum to traffic to the cell surface, but it seems more likely that most of the newly synthesized MRAP will traffic independently to the cell surface where it may partner with un-partnered MC2R, thus creating new functional receptor. Consistent with this is the observation that MRAP protein may turnover more rapidly than MC2R, although the mechanisms and cellular location for this degradation are not clear.

## Author Contributions

This work is the result of discussion between the authors. The initial draft was prepared by AC and revised by both authors.

### Conflict of Interest Statement

The authors declare that the research was conducted in the absence of any commercial or financial relationships that could be construed as a potential conflict of interest.
